# Aberrant Expression of Immunohistochemical Markers in Malignant Melanoma: A Review

**DOI:** 10.3390/dermatopathology8030040

**Published:** 2021-08-03

**Authors:** Elie Saliba, Jag Bhawan

**Affiliations:** Dermatopathology Section, Department of Dermatology, Boston University School of Medicine, Boston, MA 02118-2415, USA; esaliba@bu.edu

**Keywords:** malignant melanoma, immunohistochemistry, aberrant expression, melanocytic markers

## Abstract

Immunohistochemical stains are increasingly used to aid in the diagnosis of malignant melanoma, especially when the differentiation of the tumor is unclear based on examination with hematoxylin and eosin. However, aberrant expression of non-melanocytic markers has been reported in melanomas, which can sometimes be further complicated by the loss of conventional melanocytic markers. This review aims to summarize available data regarding unusual staining patterns in primary and metastatic malignant melanoma. It also raises awareness of the potential pitfalls and highlights the importance of appropriate use and interpretation of broad immunohistochemical markers in the context of clinical and histopathologic findings to facilitate the diagnosis of atypical cases of malignant melanoma.

## 1. Introduction

The gold standard for melanoma diagnosis is histological examination with hematoxylin and eosin (H & E). However, malignant melanoma can exhibit varied morphologic features that make definitive diagnosis challenging, as it can be mistaken for other neoplasms. Immunohistochemical stains may be employed to aid in diagnosis, especially when the differentiation of the neoplasm is unclear or when the lesion is partially sampled. In the early 1980s, S100 was first identified as a useful melanocytic marker expressing a high sensitivity approaching 100%, and it is commonly expressed in all subtypes of melanoma, including desmoplastic melanoma. A large number of other melanocytic markers have been subsequently investigated including HMB45, Melan A, tyrosinase, MITF, and SOX10, and are increasingly used in the diagnosis of melanoma [1,2,3]. Nevertheless, rare cases of malignant melanoma can present with unusual staining patterns. This includes aberrant expression of non-melanocytic markers and/or lack of expression of the commonly used melanocytic markers (Table 1).

## 2. Loss of Melanocytic Markers in Malignant Melanoma

### 2.1. Metastatic Malignant Melanoma

The immunophenotypic profile may evolve during the course of malignant melanoma, most commonly manifesting in the loss of conventional melanocytic marker expression in metastatic lesions [4]. In fact, Aisner et al. report that 1% of metastatic melanoma specimens are negative for S100. Analysis of these cases showed the time interval of S100 loss ranged from three weeks to three years. No association was found between S100 negativity and the histologic subtype or site of metastasis. A diagnosis of metastatic melanoma with a negative S100 status can be rendered by negative workup for carcinoma, lymphoma, and sarcoma in addition to positive MART-1 immunoreactivity and/or prior documentation of melanoma [4].

Additionally, Agaimy et al. reported 13 cases of metastatic malignant melanoma with complete loss of differentiation markers. All cases were negative for S100, HMB45, Melan A, and SOX10. Aberrant expression for smooth muscle actin (SMA), pancytokeratin, and desmin was also noted. The diagnosis was made after exploration of the remote history and with the help of molecular testing [5].

### 2.2. Desmoplastic Melanoma

**Figure 1:**
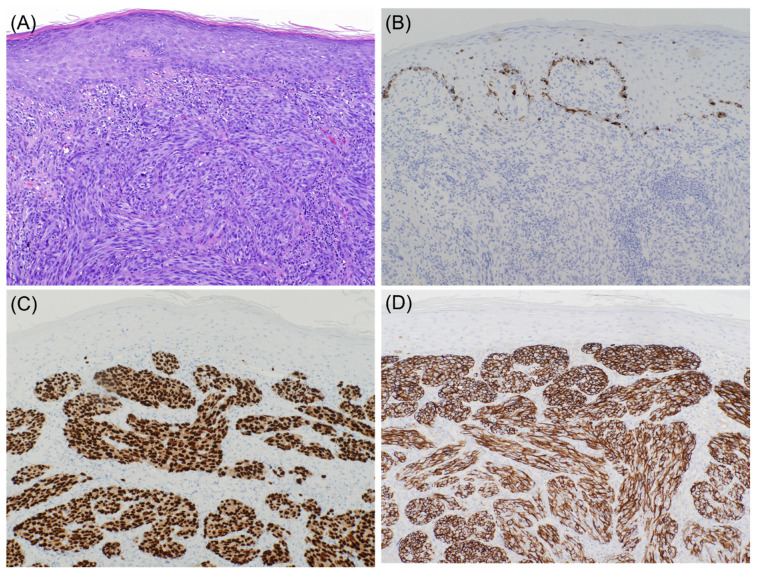
Spindle cell melanoma ((**A**), hematoxylin and eosin (H & E) stain ×10. Lesional cells are negative for Mart-1 ((**B**), immunohistochemical stain ×10) and positive for SOX10 ((**C**), immunohistochemical stain ×10) and NGFR ((**D**), immunohistochemical stain ×10).

**Figure 2:**
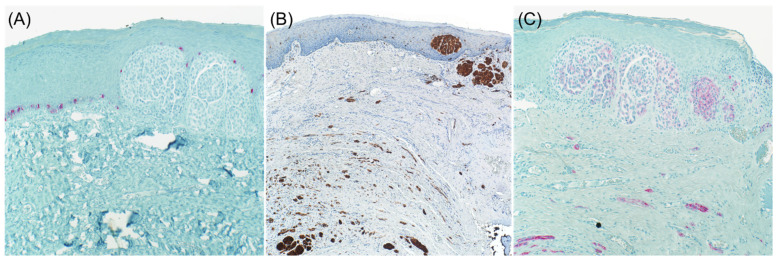
Desmoplastic melanoma. Lesional cells are negative for Mart-1 ((**A**), immunohistochemical stain ×10) and positive for S100 ((**B**), immunohistochemical stain ×4) and NGFR ((**C**), immunohistochemical stain ×10).

It is well known that desmoplastic melanoma often stains negative for specific melanocytic markers such as Tyrosinase, HMB-45, and Melan A (Figure 1 and Figure 2). S100 protein is the primary immunohistochemical stain used, with a sensitivity approaching 90% [6,7]. However, cases with focal or absent S100 staining have been reported, rendering the diagnosis of melanoma more challenging [8]. NGFR, a marker of Schwannian differentiation, has been shown to be a useful confirmatory stain for desmoplastic melanoma even when staining with S100 protein was focal or weak in many cases [8]. It should be emphasized that the expression of NGFR is not specific to neural cells as it may be expressed in basal keratinocytes, outer root sheath of hair follicles, myoepithelial cells of sweat glands, perivascular fibroblasts, and nerve fibers. As a result, NGFR should be used in combination with S100, especially in the setting of desmoplastic melanoma [9]. As NGFR was proven to be useful for the diagnosis of desmoplastic melanoma, SOX10 was discovered to be as valuable [10]. Since then, there has been a case report of SOX10 negative desmoplastic melanoma [11].

### 2.3. Primary Melanoma

Shinohara et al. reported an unusual case of primary melanoma from the right temple of a 62-year-old man with loss of staining for S100 protein, HMB-45, and Melan A, but expressed tyrosinase. Interestingly, regional lymph node metastases showed positive S100 protein, MITF, and tyrosinase staining, further emphasizing the protean nature of melanoma. The morphologic features of the tumor were of a poorly differentiated carcinoma, melanoma, or other neoplasms. Additional markers for carcinoma, vascular, and hematopoietic neoplasms were all negative. This case highlights the fact that, although tyrosinase is reported to be less sensitive than S100 protein, it is one of the most specific immunostains that may prove useful when the diagnosis of malignant melanoma is in doubt [12]. This case also showed aberrant diffuse and weak expression of CD68 in both the primary cutaneous melanoma and lymph node metastases. The authors questioned the specificity of CD68 as it can be detected in lymphomas, carcinomas, and up to 70 percent of melanomas as reported in one case series [12].

Chang and Argenyi reported another case of primary melanoma on the back of a 58-year-old man, in which the central portion of the tumor had lost expression of common melanocytic markers and expressed aberrant epithelial markers including pancytokeratin and CMA 5.2. Sentinel lymph node biopsy showed a similar cytomorphology to the primary tumor, with loss of expression of melanocytic markers, in addition to aberrant staining with PAX-8 and the epithelial markers mentioned previously. Several months later, the patient presented with a subcutaneous nodule in his right lateral hip. The excision revealed a malignant neoplasm with positive reactivity to pancytokeratin, CAM 5.2, and PAX-8, in addition to S100, SOX10, and Melan-A. Renal cell carcinoma was suspected based on PAX-8 immunoreactivity. However, no primary renal tumor was found on imaging studies. Given the similar immunophenotype as the primary melanoma and immunohistochemical evidence of melanocytic differentiation, the lesion was interpreted to be a metastatic melanoma [13].

## 3. Aberrant Expression of Non-Melanocytic Markers

### 3.1. Muscle-Specific Markers

The aberrant expression of desmin in melanoma was initially reported in a single case by Truong et al. [14]. Later on, a few cases of desmin-positive melanomas have been reported, including some exhibiting smooth or skeletal muscle differentiation [15]. Moreover, primary sinonasal mucosal melanoma exhibiting varying histologic phenotypes, including small round blue cell morphology epithelioid and focal rhabdoid morphology, was reported in a 70-year-old male. On immunohistochemistry, the tumor cells were diffusely positive for desmin, prompting a diagnosis of rhabdomyosarcoma. However, other myogenic markers were negative. Additional immunohistochemical studies revealed positivity for S100 protein, Tyrosinase, HMB-45, and Melan A and negativity for other markers including CKAE1/3, synaptophysin, chromogranin, EMA, CD99, CD45, CD3, and CD20 [16]. Rhabdomyosarcoma arising in the sinonasal tract is the major differential diagnosis to be considered. However, positive staining of rhabdoid lesional cells for melanoma markers and their negative staining for most myogenic markers such as myogenin and Myo-D1 exclude the diagnosis of rhabdomyosarcoma [16].

Osteogenic melanoma with aberrant expression of desmin was reported in a 49-year-old woman who presented with a pigmented lesion in the subungual region of her left hallux. The tumor was composed of pleomorphic atypical epithelioid and fusiform cells with focal lentiginous proliferation of large, atypical melanocytes along the dermoepidermal junction. There were also areas of osteoid matrix that were focally mineralized. Immunohistochemistry revealed expression of S100 protein and desmin. Further, 40% of the neoplastic cells were weakly positive for Melan-A, HMB45, and MITF. The patient underwent amputation of the left hallux with negative sentinel lymph node [17]. In one study, desmin expression was found in 24% of cases of melanoma, typically confined to only a small number of cells. Interestingly, aberrant desmin expression was a frequent finding in S100 protein negative melanomas, present in three of five such cases. The authors concluded that this likely represents a referral bias, as these were all consultation cases [18].

**Figure 3:**
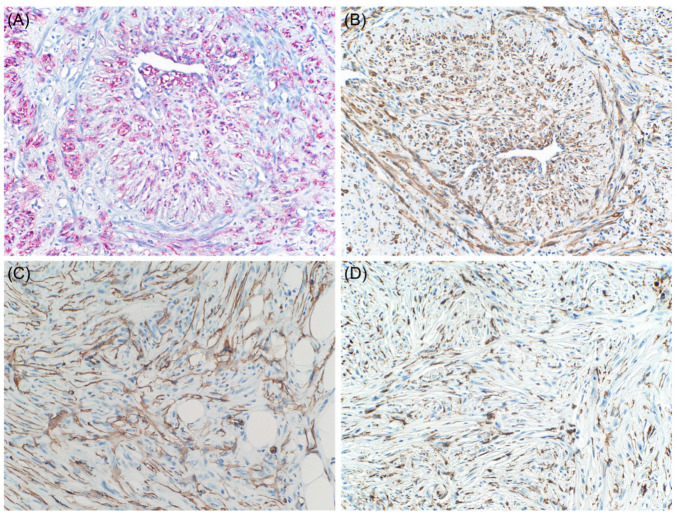
Desmoplastic and neurotropic melanoma. Lesional cells are positive for NGFR ((**A**), immunohistochemical stain ×20) with aberrant expression of actin ((**B**), immunohistochemical stain ×20), CD34 ((**C**), immunohistochemical stain ×20), and CD68 ((**D**), immunohistochemical stain ×20).

On the contrary, Banerjee and Harris reported that α-SMA positivity is extremely uncommon in conventional cutaneous or metastatic melanomas [19]. However, desmoplastic malignant melanomas may show diffuse positivity for this marker (Figure 3) [20].

Additionally, one study has shown that malignant melanoma cells release a platelet derived growth factor-like substance that inhibits the expression of smooth muscle alpha-actin and has a suppressive effect on the contractile elements in non-neoplastic cells. The study has found that, in close vicinity to the tumor, the SMA in the vessels walls was often discontinuous and obscure, and no melanoma cells were stained with SMA. These results suggest that tumor cells can release factors that alter the cytoskeletal system in the surrounding stroma [21].

Calponin is a 32 to 36 kDa protein that is associated with the cytoskeletal fraction of actin, and is identified in smooth muscle cells, myofibroblasts, and myoepithelial cells. Lee et al. reported three cases of sinonasal melanoma focally positive for Calponin. In two cases, the cells with calponin immunoreactivity appeared to be the same cells stained with chromogranin. None of these cases showed spindle cell morphology or desmoplastic reaction [22].

### 3.2. Neuroendocrine Markers

Aberrant expression of neurofilament protein and glial fibrillary acidic protein has only very rarely been reported in malignant melanoma. Romano et al. investigated the expression of these markers in 71 patients diagnosed with malignant melanoma. Here, 5/31 (16%), 3/32 (9%), and 10/34 (29%) of the cases were aberrantly positive for neurofilament protein, glial fibrillary acidic protein, and synaptophysin, respectively, confirming the prior published observations. In contrast, chromogranin A expression, which usually parallels that of synaptophysin, was absent in all cases [18]. Additionally, neuroendocrine differentiation with expression of melanocytic and neuroendocrine markers, including chromogranin, synaptophysin, neurofilament protein, and HMB-45, was reported in three cases of malignant melanoma [23].

CD56, also known as neural cell adhesion molecule, is normally expressed on neurons, skeletal muscle, and natural killer cells. However, it lacks the specificity of the neuroendocrine markers mentioned above. Aberrant CD56 expression in primary esophageal melanoma was reported in one case. The neoplastic cells also stained positive for S100, Melan A, and SOX10 and negative for cytokeratin Synaptophysin, Chromogranin, p63, TTF-1, LCA, and other lymphoid markers. A primary esophageal melanoma can cause diagnostic difficulty, particularly when a small biopsy is available for evaluation [24]. In fact, up to 25% of the cases are only diagnosed following complete resection of the tumor [25]. Therefore, a wide battery of melanocytic markers will be of help to make the correct diagnosis, keeping in mind that malignant melanoma may also aberrantly express cytokeratin and neuroendocrine markers [24].

Additionally, in a case series of 14 patients published by Steppert et al., CD56 was positive in about half of the samples. For primary melanoma, CD56 was weakly positive in 2/4 cases. For the metastases, 20 specimens were evaluated. CD56 was strongly positive in 4 cases, weakly positive in 5 cases, and negative in 11 cases. Further, 8/15 specimens also stained positive for synaptophysin. No specimen stained for Chromogranin A. Single cell positivity can be found in CD56-positive tumor infiltrating lymphocytes, but the strong positivity is considered relevant when the tumor cells themselves express CD56 [26].

### 3.3. Keratin

Keratin in melanoma was first reported by Gatter et al. The authors noted immunoreactivity with CAM 5.2 antibody in 4 of 41 fresh frozen melanomas. However, they did not find keratin expression in routinely processed melanomas [27]. These results were validated by a study that later reported similar CAM 5.2 immunoreactivity in a subset of frozen melanomas and in a smaller number of routinely processed tumors [28]. Additionally, Zarbo et al. demonstrated using Western blot that keratin-immunoreactive melanomas were truly producing keratin peptides. They were also able to find aberrant keratin expression in recurrent or metastatic melanomas [29]. Recently, one study reported aberrant expression of cytokeratin AE1/AE3 in 40% of cases and OSCAR antibody in 28% of cases. The higher frequency of keratin expression presumably reflects the use of modern epitope retrieval techniques, as well as consultation bias. In contrast to prior reports, the authors did not find any difference in the frequency of keratin expression in primary versus metastatic melanomas. The expression of keratin is, however, more common in epithelioid as compared with desmoplastic melanomas [18].

Plotzke et al. reported the expression of CK cocktail in 1.2% (3/248) of melanoma cases, only one of which was diffusely positive. All three cases were epithelioid metastatic melanomas without spindle cytomorphology. The association between cytokeratin cocktail positivity and metastasis was statistically significant [30].

Furthermore, Saggini et al. reported a case of primary intrafascial desmoplastic melanoma with aberrant cytokeratin AE1/AE3 expression, arising on the scalp of a 71-year-old man. Histologically, the tumor exhibited a biphasic pattern, with a predominating hypocellular proliferation of spindled cells and foci of polygonal to round epithelioid cells. On immunohistochemistry, both populations were diffusely positive for S100 and SOX10 and negative for HMB-45, CAM5.2, CK-7, CK-20, EMA, SMA, desmin, GFAP, TTF-1, CDX-2, and p63. Additionally, the epithelioid, but not the conventional desmoplastic part, exhibited strong staining with pan-cytokeratin AE1/AE3 [31].

**Figure 4:**
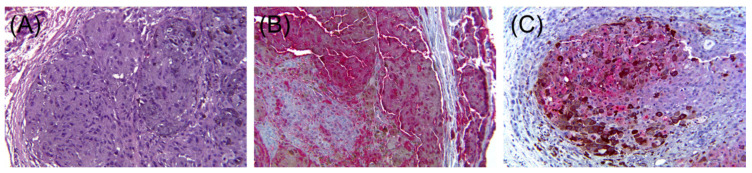
Malignant melanoma ((**A**), H & E ×20). Lesional cells are positive for S100 ((**B**), immunohistochemical stain ×20) and low molecular weight keratin ((**C**), immunohistochemical stain ×20).

In summary, the expression of epithelial markers in melanoma is not uncommon, and it appears to be more frequent in cases with epithelioid cytomorphology (Figure 4). However, the expression of keratins should be carefully interpreted as, in many instances, the positivity can be only present in the surrounding stroma. These cases can be easily mistaken for carcinomas if the stain is misinterpreted as positive.

### 3.4. Macrophage Markers

Aberrant CD68 expression was reported in one case of primary amelanotic melanoma in ascites fluid of a 67-year-old man. The strong expression of CD68 and the absence of brown pigment were unusual. Negativity for epithelial markers including CK5/6, CK7, CK20, p63, CDX2, TTF1, PSA, and GATA3 helped to exclude the possibility of a carcinoma. Tumor cells were found positive for S100 and negative for non-epithelial markers including CD3, CD20, CD30, MUM1, and HHV8. In order to distinguish fibrohistiocytic tumor from malignant melanoma, immunostaining for CD163 and clusterin, together with melanocytic markers, was used. Strong nuclear SOX10 expression and a moderate reaction for Melan A were observed. Negative immunostaining for CD163 and langerin was also helpful to exclude histiocytic lymphoma and Langerhans cell Histiocytosis [32]. In summary, metastatic malignant melanoma in ascites fluid is diagnosed after immunohistochemical exclusion of carcinoma and lymphoma.

Another case of malignant melanoma with loss of conventional melanocytic markers except Tyrosinase showed immunostaining in both the primary lesion and lymph node metastasis [12]. CD68 immunostaining can occur in 75% of metastatic malignant melanoma [33]. However, these cases usually show strong nuclear immunoreactivity for SOX10, whereas fibrohistiocytic and hematopoietic neoplasms lack this reaction [33].

Shah et al. reported that CD68 is consistently expressed in primary conventional, desmoplastic, and metastatic melanomas, but negative for neural tumors and for dermal component of compound nevi, making it a useful marker for the diagnosis and differential diagnosis of such neoplasms [34].

Jensen et al. reported expression of CD163 in 35% and CD68 in 10% of 190 melanomas. It is interesting to note the CD68 expression was independently associated with poor relapse-free survival and CD163 expression was an independent prognostic factor along with tumor thickness [35].

### 3.5. Vascular Markers

**Figure 5:**
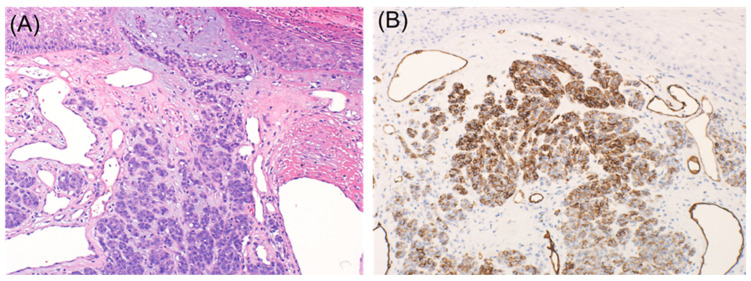
Malignant melanoma ((**A**), H & E ×10) with aberrant expression of CD31 ((**B**), immunohistochemical stain ×10).

The induction of an adequate tumor vasculature in response to the increasing demand of oxygen is important for tumor growth and invasion. Melanoma cells are thought to induce vasculogenic mimicry, which refers to the tumor cell’s ability to express endothelium-associated genes and form a vasculogenic-like patterned network [36]. Aberrant expression of vascular markers such as CD31 and CD34 has been reported in malignant melanoma (Figure 5).

Pisacane et al. demonstrated the immunohistochemical expression of CD31 and CD34 in 22/30 (73%) and 10/30 (33.3%) of invasive cutaneous melanomas cells, respectively. CD31, but not CD34 also stained 10 of 15 metastatic melanomas. Additionally, CD31 and CD34 expression was never found in common melanocytic nevi. The authors also found that CD31 and CD34 expression closely parallels the morphologic phases of melanocytic tumor progression corresponding to Clark’s levels and their associated metastatic potential [36].

Salven et al. also reported that VEGF is upregulated during the course of melanoma progression. The study showed that lesional cells are significantly more likely to stain for VEGF in metastatic versus primary melanomas (91% versus 32%, respectively). Tumor infiltrating inflammatory cells also expressed VEGF in all melanomas [37].

CD34 has been described as a marker of dermatofibrosarcoma protuberans (DFSPs). Its expression has also been documented in benign neural tumors including neurofibroma and schwannoma, and spindle cell neoplasms such as solitary fibrous tumors and giant cell fibroblastoma [38]. CD34 expression in desmoplastic melanoma was also reported in a 72-year-old man. Clinically, the patient presented with a red lesion on the right lower abdomen. The tumor cells, which were spindled, exhibited nuclear pleomorphism and were mitotically active. They strongly expressed S100 with focal expression of Tyrosinase. A diffuse strong positivity for CD34 by spindle neoplastic cells was also noted [38]. Breza and Margo also reported another case of primary cutaneous melanoma with strong expression of CD34 and S-100 and focal positivity for Melan-A and HMB-45 [39].

### 3.6. Hematopoietic Markers

Expression of hematopoietic markers in non-hematopoietic neoplasms is extremely uncommon. Chatzopoulos et al. observed a strong tumor cell membranous reactivity for CD4 in two cases of metastatic melanoma. The authors considered the hypothesis of CD4+ lymphocytes phagocytosis by melanoma tumor cells with resulting immunoreactivity or even the possibility of an artifactual cross-reactivity due to endogenous Fc receptor expression by tumor cells. The exact significance and mechanism of CD4 expression are unknown and remain to be elucidated by further studies [40].

Fang et al. identified a small subpopulation of melanoma cells with stem cell-like properties that express CD20, but are negative for CD3, CD4, CD8, and CD45. Furthermore, these cells were more tumorigenic than adjacent cells when grafted to mice. It is possible that this aberrant expression of hematopoietic cells may allow melanoma cells to easily access the circulation and establish distant metastases [41].

### 3.7. FLI-1

FLI-1 nuclear transcription factor has been proposed as a tool in the differential diagnosis of small round cell sarcomas. It has also been described as a marker in endothelial differentiation. Expression of FLI-1 has been demonstrated in Ewing’s sarcoma/primitive neuroendocrine tumor, small cell carcinomas of the lung, and benign and malignant vascular neoplasms. Strong expression of FLI-1 has been reported only in seven cases of melanoma that displayed variable expression of melanocytic markers [42]. Overall, FLI-1 expression was higher in metastatic melanoma than in primary tumors and was associated with aggressive behavior [42,43,44]. Interestingly, one case of metastatic melanoma with loss of conventional markers expressed FLI-1 diffusely. The diagnosis was confirmed with BRAF V600E immunohistochemistry as this mutation had been identified in one of the patient’s prior metastatic lesions. Strong and diffuse expression of FLI-1 in melanoma in the setting of metastasis with unusual morphology could be deceiving. Correlation with clinical and previous molecular findings is paramount for the correct diagnosis of these cases [42].

### 3.8. GATA3

GATA3 is commonly used as a marker for mammary and urothelial carcinoma, especially in the setting of distinguishing the origin of metastatic carcinoma. However, it is not entirely specific as it can also be expressed in skin tumors including both epidermal and adnexal neoplasms, but not in melanoma [45]. Metastatic melanoma with aberrant expression of GATA3 and focal weak expression of pan cytokeratin was reported in one case report. The patient is a 52-year-old woman with a previous history of malignant melanoma and breast cancer who presented for a left shoulder mass. Pathologic examination showed poorly differentiated tumor cells with anaplastic morphology and extensive necrosis. The tumor cells were strongly immunoreactive for GATA3 with focal weak expression of pan cytokeratin. Six melanocytic markers were performed including S100, Melan A, HMB45, SOX10, MITF, and Tyrosinase, and were all negative. BRAF V600K mutation was demonstrated in the newly developed lesion, confirming the diagnosis of metastatic dedifferentiated melanoma [45]. Recognition of melanoma is important to avoid misdiagnosis of carcinoma, especially with the history of breast cancer and the expression of GATA3 by tumor cells, which is usually used as a marker for mammary carcinoma. Clues that suggested the diagnosis in this case included the correlation with previous melanoma cytomorphology, knowledge about the previous history of melanoma, and cytogenetic testing for known mutations [45].

### 3.9. CEA

**Figure 6:**
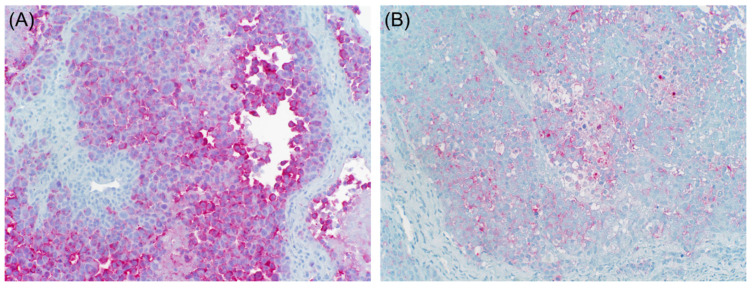
Malignant melanoma. Lesional cells are positive for Mart-1 ((**A**), immunohistochemical stain ×20) with aberrant expression of CEA ((**B**), immunohistochemical stain ×20).

CEA immunoreactivity was shown to be expressed in 15 of 28 cases of metastatic melanoma when polyclonal antibodies were used. No staining was seen with monoclonal anti-CEA antibodies [46]. A study conducted by Sanders et al. revealed that 14 of 20 (70%) cases of superficial spreading melanomas expressed CEA, which was demonstrated in junctional and intradermal melanoma cells. Six of ten (60%) nodular melanomas showed focal strong membranous and/or weak cytoplasmic staining. The same pattern of positivity was also seen in seven of nine skin metastases and two of three lymph node metastases. The study highlights the fact that CEA expression can be seen in a large percentage of primary and secondary malignant melanomas (Figure 6) [47].

Another study reported all 12 cases of primary melanoma to express polyclonal CEA, while only 1 of 11 Spitz nevi was positive for this marker, highlighting the potential for this antibody to distinguish between these two entities [48].

### 3.10. Calretinin

Calretinin is a calcium binding protein that is structurally related to S100 protein and inhibin. Immunohistochemical stain for calretinin shows nuclear and cytoplasmic staining in mesothelial cells, adipocytes, endometrial stromal cells, ganglion cells, Sertoli cells, and Leydig cells. Aberrant staining for calretinin stain is rare in melanoma [49]. Metaplastic melanoma with aberrant expression of calretinin stain in both the chondroid component and malignant cells adjacent to it was described in one case report.

Metaplastic melanoma has not been previously reported to stain for calretinin. Rare incidents of calretinin positive melanoma are reported in epithelioid, pleomorphic, and metastatic melanoma. The awareness of the possibility of aberrant calretinin positivity in metaplastic melanoma with chondroid differentiation is critical to avoid a potential pitfall in misdiagnosing metaplastic melanoma as sarcoma or mesothelioma [49].

### 3.11. PAX8 and PAX2

PAX8 and PAX2 are common markers for renal or Mullerian differentiation. While most PAX8+ and PAX2+ carcinomas are rarely confused with melanoma, cases of MiTf family altered renal cell carcinoma (MiTF-RCC) may pose a diagnostic challenge owing to the frequent patchy expression of melanocytic markers. In a study of 263 melanomas, the expression of PAX8 was reported in 7.9% of cases and was significantly associated with spindle cytomorphology. PAX2 was positive in one (0.4%) case. In summary, caution should be exercised when evaluating a skin tumor in patients with history of MiTF-RCC as a small subset of primary or metastatic melanomas may demonstrate PAX8 or PAX2 staining. Ultimately, these markers should be used in a broad immunohistochemical panel rather than in isolation, in addition to molecular or cytogenetic studies when in doubt [30].

## 4. Conclusions

In conclusion, malignant melanoma showing aberrant expression of immunohistochemical markers may easily be mistaken for carcinomas, lymphomas, sarcomas, and neuroendocrine tumors. Luckily, the majority of these cases showing aberrant expression will stain positive for at least one specific melanocytic marker. Awareness by pathologists of these potential pitfalls, and appropriate use and interpretation of broad immunohistochemical markers together with the clinical history, should facilitate the diagnosis of malignant melanoma with unusual staining patterns.

## Figures and Tables

**Table 1:** Aberrant expression of immunohistochemical markers in malignant melanoma.

Loss of Melanocytic Markers
Primary melanoma including desmoplastic
Metastatic melanoma
**Aberrant Expression of Non-Melanocytic Markers**
Muscle-specific markers
Desmin and α-SMA
Calponin
Neuroendocrine markers
Neurofilament protein, glial fibrillary acidic protein, synaptophysin, and chromogranin
CD56
Keratin
CK AE1/AE3, OSCAR, and CAM 5.2
Macrophage markers
CD68 and CD163
Vascular markers
CD31 and CD34
Hematopoietic markers
CD4
CD20
Miscellaneous
FLI-1
GATA3
CEA
Calretinin
PAX8 and PAX2
